# TheHiveDB image data management and analysis framework

**DOI:** 10.3389/fninf.2013.00049

**Published:** 2014-01-06

**Authors:** J-Sebastian Muehlboeck, Eric Westman, Andrew Simmons

**Affiliations:** ^1^Department of Neuroimaging, Institute of Psychiatry, King’s College LondonLondon, UK; ^2^Department of Neurobiology, Care Sciences and Society, Karolinska InstitutetStockholm, Sweden; ^3^J-S Muehlboeck Inc., MontrealQC, Canada; ^4^NIHR Biomedical Research Centre for Mental Health, King’s College LondonLondon, UK; ^5^NIHR Biomedical Research Unit for Dementia, King’s College LondonLondon, UK

**Keywords:** neuroimaging database framework, image processing, query interface, data management, data query, neuroimaging collaboration and workflows, web 2.0 application

## Abstract

The hive database system (theHiveDB) is a web-based brain imaging database, collaboration, and activity system which has been designed as an imaging workflow management system capable of handling cross-sectional and longitudinal multi-center studies. It can be used to organize and integrate existing data from heterogeneous projects as well as data from ongoing studies. It has been conceived to guide and assist the researcher throughout the entire research process, integrating all relevant types of data across modalities (e.g., brain imaging, clinical, and genetic data). TheHiveDB is a modern activity and resource management system capable of scheduling image processing on both private compute resources and the cloud. The activity component supports common image archival and management tasks as well as established pipeline processing (e.g., Freesurfer for extraction of scalar measures from magnetic resonance images). Furthermore, via theHiveDB activity system algorithm developers may grant access to virtual machines hosting versioned releases of their tools to collaborators and the imaging community. The application of theHiveDB is illustrated with a brief use case based on organizing, processing, and analyzing data from the publically available Alzheimer Disease Neuroimaging Initiative.

## INTRODUCTION

The advent of increasing numbers of large longitudinal imaging studies, imaging-genetics studies, and multi-center studies and the need to curate large volumes of imaging data from individual studies for data reuse purposes has led to a growing need for an integrated brain imaging database, resource, data, and activity management system. A number of imaging databases have been described in the literature including the LONI IDA (Van Horn and Toga, 2009), Loris (Das et al., 2012), and XNAT (Marcus et al., 2007) systems. Each of these databases represent attempts to create a system capable of jointly managing the increasing amounts of imaging data and data from other sources and modalities, while providing support for the specific processing requirements of imaging projects. They have been created in and for very specific environments with their own respective emphases and limitations.

The driver for the creation of a new alternative approach arose from a series of joint studies between King’s College London, the Karolinska Institute, and our collaborators working on a number of large imaging studies including AddNeuroMed (Lovestone et al., 2007, 2009), Alzheimer Disease Neuroimaging Initiative (ADNI; Jack et al., 2008; Weiner et al., 2010), and AIBL (Ellis et al., 2009). The hive database system (theHiveDB) has been developed to match requirements not easily reconciled with the alternatives mentioned above. TheHiveDB offers a consistent solution to the intricacies of imaging projects. For ongoing projects and pre-existing collections of data it provides viable approaches to properly organize, manage, and store, both imaging and associated non-imaging data types. It is first and foremost a data aggregation and management system with a focus on easy interactions with the researcher.

## MATERIALS AND METHODS

### NEUROIMAGING PROJECT CHARACTERISTICS

Imaging projects consist of sets of participants, referred to here as individuals, typically divided into different groups (e.g., patients and healthy controls, or those who respond/don’t respond to the effects of a novel drug). According to study protocols individuals may present on a number of occasions. These might be visits for cognitive tests or scanning sessions. Once data is acquired it is assigned to predefined labels (e.g., Baseline, 1-year-follow-up etc.), referred to here as timepoints. Imaging data is acquired in conjunction with a plethora of clinical, behavioral, and genetic data. Data from these modalities are frequently available in tabular form and often need to be combined across modalities for subsequent analysis. To properly support imaging data a neuroimaging database framework also needs to support the management of binary files, which we refer to as assets. We will consider here a use case of magnetic resonance imaging data, though the system is designed flexibly so that PET, SPECT, digital X-rays, or other medical images can also be managed.

At each timepoint a study consists of a series of images (for example a MRI localizer, multi-slice T2-weighted fast spin echo images and a T1-weighted ultrafast gradient echo volume). Each individual series will often consist of a number of slices or volumes. To guarantee usable and comparable results, scanning protocols are often pre-defined and matched to other imaging studies. TheHiveDB is designed to manage and organize raw and processed imaging data in conjunction with other available data such as demographic, cognitive, biological sample, and genetic data. Special attention and support are given to raw imaging data which is efficiently archived, properly stored and thoroughly documented. Image types can be defined by means of scanning protocol parameters, such that image assets can be extracted automatically from raw data archives. The system provides image format conversion routines for the resulting image assets. Assets are accessible to authorized users (project members) through a web interface via secure streaming. Tabular data can be downloaded through an interactive query interface.

For image processing, an activity component allows the execution and automation of frequent imaging tasks or application of standard image processing pipelines by means of a convenient web interface. Activities are defined in terms of the required inputs and resources designated to carry them out. Activity instances can be created by resource owners and assigned to projects.

For effective network management and security the authentication, authorization, and accounting (AAA) architecture has been chosen. System users need to authenticate to access the system. The authorization function is split into access to file data (assets), which is granted by means of project memberships (and is possible via the web interface) and user roles. The latter define the extent to which a user can interact with the system (e.g., only query data versus upload data and request processing). The activity system provides tracking and accounting functionality.

The main aspects of the system are:

• Asset management – storage, data archival, retrieval/access, availability, transfer, backup.

• Data processing – rendering algorithms available and usable for projects in an automated and traceable fashion.

• Resource management and sharing – to reduce overhead and cost, existing resources can be managed effectively and shared efficiently.

• Data querying – interactive querying of variables of interest across modalities.

### APPLICATION ARCHITECTURE

The Hive database web application has been developed using the Grails open source web application framework based on the Groovy programming language. Groovy is an object-oriented programming language for the Java platform, which is dynamically compiled to java virtual machine (JVM) byte-code. Since most Java code is also syntactically valid Groovy code it interoperates seamlessly with existing Java code and libraries. The Grails framework interacts with relational database engines using object relational mapping. Hibernate^1^ is used for relational persistence. MySQL has been chosen as the default database for theHiveDB due to its performance, wide-spread availability, transactional support, and web and data warehouse strengths^2^. As a full stack web application framework Grails provides performance optimized layers for communication with the back end, domain object mapping, database communication, and caching. Our current production environment hosts imaging for about 18,000 scanning sessions with 50,000 series and over 33 million documented DICOM files. The entire application is connected to various profiling utilities to identify and address scenarios where response times for the web interface are above 800 ms.

TheHiveDB relies on job scheduling^3^ for any request likely to use significant CPU resources (e.g., run Freesurfer or DICOM archive creation). For these requests a job record is created with instant feedback to the user. The same applies for data transfers. The system handles jobs and transfers independently of the user’s session based on resource availability, priorities, and concurrent requests.

**FIGURE 1:**
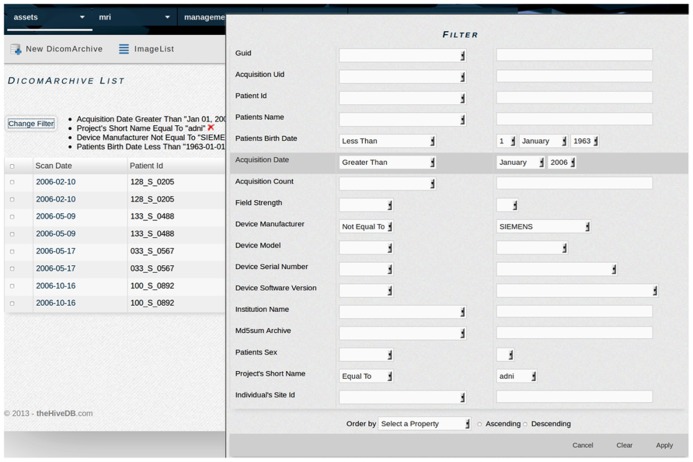
**TheHiveDB provides extensive filters for searching within entity lists (e.g., individuals or different types of assets).** The example shows a filter used for searching ADNI DICOM archive data using specific criteria like data acquired for individuals born prior to 1963 and scanned after January 2006 on a non-Siemens scanner.

The web application interface is accessible via secure http (https), which provides bidirectional encryption of communications between client and server. The system communicates with all resources using a pure Java implementation of the SSH-2 protocol^4^. The web interface relies heavily on JavaScript libraries to enhance the user’s experience. JavaScript libraries are used within the context specific help system, data filters (see **Figure 1**), and the dynamic query interface. Additionally some views have JavaScript enhancements to allow for viewing adjustments.

The activity system extensively uses the open source grid engine [formerly Sun grid engine (sge)] for job scheduling, monitoring and resource management. Grid Engine is software that facilitates “distributed resource management” (DRM). Far more than just simple load-balancing tools or batch scheduling mechanisms, DRM software typically provides the following key features across large sets of distributed resources^5^:

• Policy based allocation of distributed resources (CPU time, software licenses, etc.)

• Batch queuing and scheduling

• Supports diverse server hardware, operating systems (OSs), and architectures

• Load balancing and remote job execution

• Detailed job accounting statistics

• Fine-grained user specifiable resources

• Suspension, resumption, and migration of jobs

• Tools for reporting Job/Host/Cluster status

• Job arrays

• Integration and control of parallel jobs

The integration of other job schedulers within theHiveDB is feasible as long as they support the features listed above.

### STORAGE ARCHITECTURE

TheHiveDB facilitates the work of research groups by offering a unified approach to management, sharing, and processing of imaging data research projects. It has been designed as an imaging project and data management system with an integrated activity component. Imaging projects are created using the web interface. Study participants (individuals) are assigned to projects using project specific identifiers. Individuals can be created and maintained through the web interface, direct upload of individual lists or automatically derived from DICOM^6^ header data.

**FIGURE 2:**
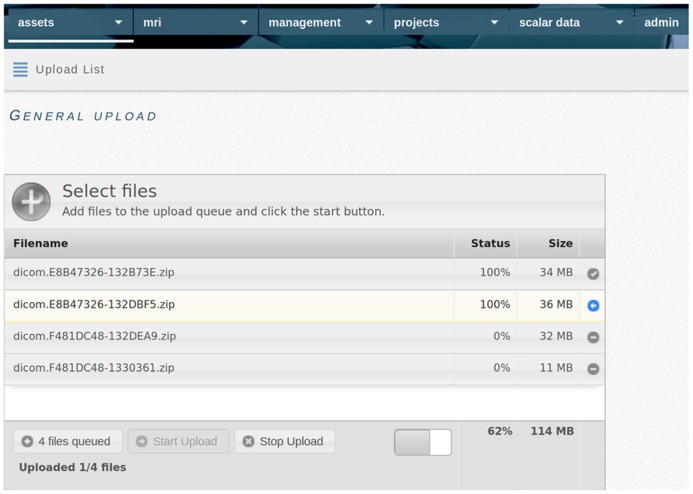
**TheHiveDB features a web based upload interface, which allows local data to be uploaded to the database.** The multi file upload allows for drag and drop and shows upload progress.

**FIGURE 3:**
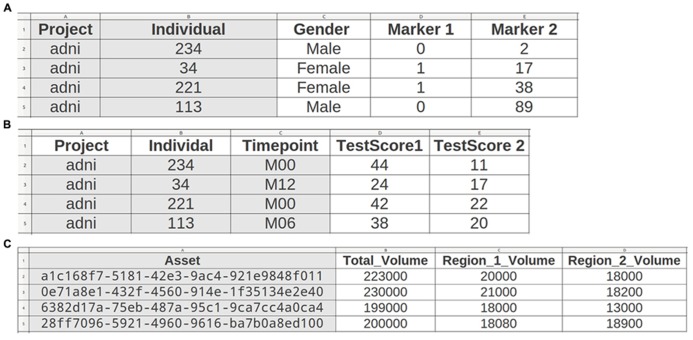
**TheHiveDB supports convention based import of tabular data.** Scalar data can be imported on three levels: describing individuals **(A)** (e.g., gender, genetic data), individuals at timepoints **(B)** (e.g., clinical tests), or assets **(C)**. Since the asset belongs to a project, individual, and timepoint (e.g., activity output) the assignment can be performed automatically by just providing the unique asset ID.

All file data enters the system through a web-based upload interface (**Figure 2**). File naming conventions and manual assignment can be used for allocation to projects. Uploaded tabular data is incorporated directly (e.g., individual list or cognitive test result; see **Figure 3**), while (binary) files are recorded as assets. Assets are data entities managed by theHiveDB. They are registered upon creation or upload and can be transferred for processing or downloaded via streaming through the web interface. Every asset belongs to a project, individual, and timepoint by virtue of being assigned to it directly (e.g., an image) or by inheritance (e.g., an image transform, the modified representation of an image outputted by an image processing algorithm).

To manage assets effectively theHiveDB relies on predictable unique identifiers. TheHiveDB automatically computes and assigns such identifiers to all newly created assets. The identifiers are predictable, because they are determined based on information about the actual asset or the process leading to its creation. Technically the identifier is a deterministic universally unique identifier (dUUID). A UUID is a 16-octet (128-bit) number. In canonical form, it is represented by 32 hexadecimal digits, displayed in five groups separated by hyphens for a total of 36 characters (8-4-4-4-12, i.e., 32 alphanumeric characters and four hyphens, e.g., 6d0b1c00-2a11-4aaa-a337-3ba06e9ee2ef). UUIDs are frequently used in distributed systems to uniquely identify information. A UUID by itself is not human interpretable. Within theHiveDB however it is used as a powerful alias for the asset it refers to. TheHiveDB web interface offers the possibility to use UUIDs like tracking numbers and will assemble details for all assets listed in the search field. User preferences govern how assets are renamed for the individual user upon download. If the above example for instance refers to a DICOM archive, the user may choose to retrieve such files as managed by the system (i.e., 6d0b1c00-2a11-4aaa-a337-3ba06e9ee2ef.tar), identified for asset type (i.e., dicomArchive.6d0b1c00-2a11-4aaa-a337-3ba06e9ee2ef.tar), enriched with human-interpretable information (e.g., DCM.AcquisitionDate.PatientID.6d0b1c00-2a11-4aaa-a337-3ba06e9ee2ef.tar), etc. Similar renaming options are available for other asset types.

Aside from warranting uniqueness, predictability is another concern. Therefore within theHiveDB UUIDs are not assigned randomly, but computed in a deterministic fashion. For instance DICOM header information is used to compute identifiers for image assets. Identifiers for output from image processing algorithms or pipelines are computed taking the algorithm’s name, version, and input file identifiers into account. Consequently, requesting extraction of images from a DICOM archive containing a subset of already extracted data will result in a UUID collision. Similarly, the request to reprocess data with the same algorithm without removing previous results will fail. While there is currently no plan to implement federated searches, data exchange, or migration between HiveDB instances is planned.

Typically assets will have at least one “asset file” – the data file on disk associated with it. These asset files may exist at multiple locations (e.g., one in project space and another one as backup in the cloud).

Being tailored to the specific needs of brain imaging projects the system extends the notion of asset to a number of special assets like DICOM archives (see section “DICOM management, storage, and compression”), images (see “Images” section), output collections, and image transforms (see “Workflow” section), but can also store and manage new types of assets, as defined by the user. For example binary data files obtained from a proprietary device or program, or items with no file data like a blood sample stored in a fridge. The UUID could then be used for barcode generation.

Since images are a special type of asset with extended feature support, image files may exist in various image formats, for example DICOM and NifTi^7^. Image assets are traced and recorded as to their whereabouts just like any other asset, but in addition they can be viewed, rated, converted to other image formats, and processed using image processing algorithms.

The program md5sum is used extensively throughout theHiveDB. Md5sum is designed to verify data integrity using the MD5 (Message-Digest algorithm 5) 128-bit cryptographic hash. MD5 hashes can confirm both file integrity and authenticity. Md5sum information is registered for all assets managed by the database to allow for data verification upon transfer or backup creation.

In summary, assets are either created by directly uploading files via the web interface (see **Figure 2**) or by invoking activities on other assets already in the system. Assets specific to imaging projects extend the feature set of regular assets and the system provides built-in activities to derive, manage, and transform them effectively.

### ARCHIVING AND AUTOMATION

#### DICOM management, storage, and compression

The system supports DICOM data management by means of special assets called DICOM archives. Uploaded DICOM data is packaged and compressed after relevant DICOM header information is automatically extracted. The compression ratio (uncompressed/compressed) for the lossless compression method used is around three, resulting in space savings of about 70%. Lossless compression techniques ensure that the original data can be exactly reconstructed from the compressed data. The resulting DICOM archive assets are single files containing some metadata and the entire collection of DICOM files. Once created, DICOM archives are considered immutable. Image series can be extracted as needed without any modification to the archive. Due to the deterministic nature of the unique identifiers used, they can also be migrated and imported into other HiveDB instances.

During the archival process information about all individual DICOM series is extracted and later used for automatic validation of scanning protocols. Each individual file contained in the archive is documented as a member of a DICOM archive and DICOM series including its md5sum. The information stored in the database is a reflection of the actual data found in the DICOM headers.

Metadata is stored in the database using three data domains:

1. DICOM archives – documenting the actual archive as packaged on disk.

2. DICOM series – documenting specific parameters of individual series contained in the archive.

3. DICOM files – documenting every single DICOM slice as members of the above series and archive.

Advantages of this approach include:

• Single archives instead of thousands of files on disk per study (scanning) session, resulting in significantly improved transfer speeds and file system performance.

• Significant space savings (up to 70%).

• Convenient for long time cloud storage in Amazon Glacier or offline tape storage for backup purposes (http://aws.amazon.com/glacier/).

• Content querying and information about study available through the database instead of interaction with data on disk.

• It is possible to target individual series for extraction or conversion to various image formats.

• Data verification and validation can be performed at various levels as md5sums are stored for every single DICOM file and the entire archive.

• Regardless of original scanner export convention, files can be re-organized and fed to processing pipelines in an automated fashion (The system knows which individual files make up a series, which one is the first DICOM file, etc.).

• Data provenance. The system also extracts and manages information about the scanning device used to acquire images (e.g., Manufacturer, software version, field strength, etc.). Scanners are managed using their serial numbers and software versions, such that users can search for data acquired on specific devices.

#### Raw (DICOM) data de-identification/anonymization

Modern imaging systems conforming to DICOM specifications sometimes include protected health information (PHI) in the exported data. Privacy laws such as the European Commission’s Directive on Data Protection and the U.S. Health Insurance Portability and Accountability Act (HIPAA) restrict the sharing of data containing PHI. These laws protect citizens but complicate the day-to-day operations of scientific collaboration. Prior to any analysis of research group data or collaboration with other groups imaging data needs to be anonymized. Without a stringent workflow, users might forget to de-identify data, or incompletely de-identify data, before using it for an analysis or even sharing it. TheHiveDB enables projects to follow privacy laws affecting medical research projects. DICOM header information of newly inserted data will be visible only to the uploading user and must be confirmed as anonymized, before data can be assigned to a project. TheHiveDB is designed to coexist with PACS systems and is by no means a replacement for a PACS system. In the workflow describing a typical imaging study theHiveDB situates itself right after either an imaging system such as a MRI system or a PACS system (unless data is available publicly or via collaborators). Network architecture and local data retrieval regulations govern the interaction of theHiveDB with PACS systems. For instance, newly acquired data still located on a PACS system can either be exported and directly uploaded via theHiveDB’s upload interface or pushed to a workstation, which is registered as a HiveDB resource. PACS systems are governed by local (hospital) laws and governmental regulations. In hospital environments they may also store data for all scanned individuals, even those not to be retrieved for imaging research projects. TheHiveDB is designed to be used only with de-identified data and can easily be integrated into existing environments to enhance patient confidentiality by means of imposing a stringent workflow and data flow.

#### Images

Once a DICOM archive is assigned to a project, individual and timepoint, data becomes available and will be visible to all approved members of that particular project. Acquisition protocol details (e.g., echo time, repetition time, or slice thickness) for each project can be defined through the web interface, such that matching acquisitions can be extracted automatically as MR image assets. The system performs automated control of compliance with acquisition protocol details defined for any given project, as by default it rejects the extraction of acquisitions using invalid scanning parameters.

The image asset is an abstract entity representing the series of a certain scan type (i.e., T1 or T2 weighted MRI, etc.) obtained in the scanning session. The image asset inherits project specific properties during extraction from the original DICOM archive. As discussed in the “Storage Architecture” section it may be represented by actual files stored on disk (i.e., image files), at possibly various locations and a number of file formats. Currently a DICOM series may be extracted from the archive and stored as zipped DICOM data, compressed NifTi and minc^8^. Project settings determine which formats will be generated during the extraction. Image format conversion is part of the core system. Freely available converters will be added to produce additional image formats. TheHiveDB considers DICOM as the source for all conversions for native files, but will support conversion between formats if converters are available.

### DATA ACCESS, PERMISSIONS, AND OWNERSHIP

Users of the theHiveDB gain access to project data by means of project memberships. Projects are collections of imaging and associated data acquired or assembled with intent to answer scientific questions. Project data is stored on resources (i.e., project servers) assigned to them. Projects have administrators authorized to grant membership to new users. Upon login users may activate any number and combination of projects they are members of, to view, add, and query data or perform quality control on both original images as well as processed output (see “Annotation” section) or request activities for assets (i.e., initiate processing on imaging data). Predefined user roles determine which actions users may perform for projects (e.g., view, create, and delete assets). A user may be allowed to only view and query data, but cannot be barred from viewing access to individual assets or specific variable collections.

**FIGURE 4:**
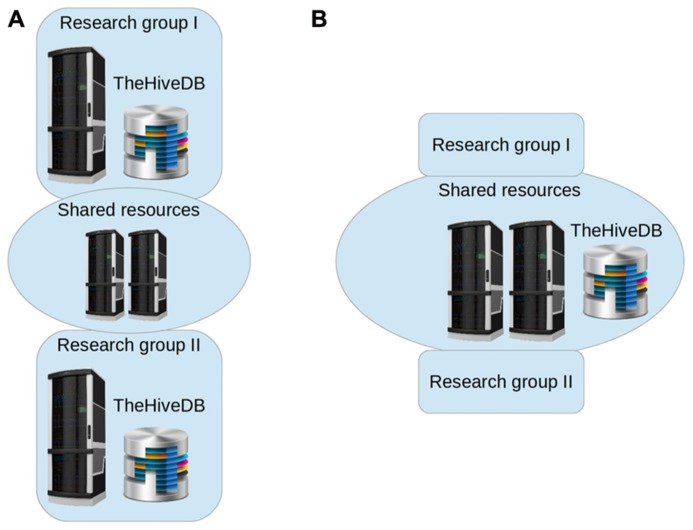
**TheHiveDB topology can be adjusted to individual requirements.** Illustration of separate database instances per research lab and some compute resource sharing [**(A)** to the left] versus collaborative setting with unified architecture [**(B)** to the right].

Each institution will have its own limitations as to available resources and project specific restrictions. TheHiveDB accommodates this variability by letting users define where data should be located and processed. A HiveDB instance may exist on a private local network only reachable through VPN or entirely rely on cloud offerings. Constraints are defined by institutional regulations, data usage, and ownership restrictions possibly on a per project basis. Groups may even choose to have two instances of theHiveDB to separate internal and collaboration databases. **Figure 4** shows topology examples for different requirements. Frequently research labs will prefer to have their own HiveDB instance with the project source data stored on an in house file system (**Figure 4A**). Collaboration in these cases will be based on sharing resources for processing purposes and possibly sharing access to subsets of projects across labs. Note, that theHiveDB database server, file servers for data store, and processing resources may well all be at different physical locations and on different servers. If however, resources are to be pooled, a unified topology arrangement (**Figure 4B**) is also a viable option.

### NEUROIMAGING DATA PROCESSING ALGORITHMS, PACKAGES, AND LIBRARIES

TheHiveDB incorporates a growing number of mechanisms for data management, archival, extraction of images, and image transforms. Additionally freely available activities and pipelines are being integrated. A range of powerful neuroimaging pipelines exist today such as Freesurfer^9^ and FSL^10^. Freesurfer will be used as an example application here. Briefly, the Freesurfer pipeline can be used for volumetric segmentation, cortical surface reconstruction, and cortical parcellation (Fischl et al., 2002, 2004). The procedure automatically assigns a neuroanatomical label to each voxel in an MRI volume based on probabilistic information automatically estimated from a manually labeled training set. This segmentation approach has been used for multivariate classification of AD and healthy controls (Westman et al., 2011a,c), neuropsychological-image analysis (Liu et al., 2010c, 2011), imaging-genetic analysis (Liu et al., 2010a,b), and biomarker discovery (Thambisetty et al., 2010, 2011).

TheHiveDB also provides convenient mechanisms for proprietary (or not publically accessible) processing algorithms to be integrated with their respective authors. Access is granted by these authors within the context of collaborative efforts. Any compute resource capable of ssh-2 connections can be registered in theHiveDB and used to perform tasks for specific projects (see “Data Processing and Workflow” section).

Activities may be triggered by project settings or via the application web interface. The transfer of required input files to available resources is performed automatically using the ssh-2 protocol for secure connections. Any activity requested by the system is logged and visible through the web interface job management module which provides live job queue monitoring, accounting and statistics. Upon job completion automated retrieval of processing output (e.g., output images and summary measures such as volumes and thicknesses) is also triggered by the job module.

TheHiveDB supports a number of common image management and processing activities directly. It supports external activities indirectly by automatically transferring required input files onto suitable resources and generating unique output collection identifiers for expected results. Upon completion of external processing these identifiers may be used to upload the results following naming conventions. For instance, a user experimenting with a new algorithm combining information from two types of MR images could register that activity and a resource for required input files. The system will then compute a unique identifier for each requested task and create a directory structure using these identifiers and place the required input files at the remote location within the respective directories. Jobs will be marked as completed once the user uploads properly named output files (i.e., using the identifiers computed for the task). With this method virtually any activity (including those requiring manual interaction) can be performed on existing assets (e.g., images) while output and results remain fully traceable.

For activities not yet to be registered and experimental purposes, assets can be pushed to any location registered by the requesting user for convenient examination or processing.

### VISUALIZATION/ANNOTATION

**FIGURE 5:**
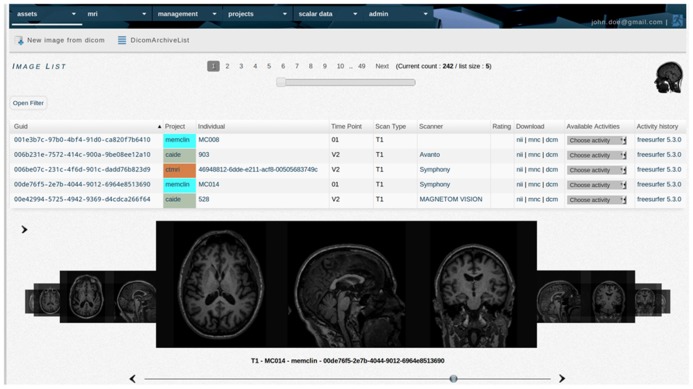
**TheHiveDB image library provides access to the quality control interface and allows the user to request processing of images.** List items are links to individuals, actual image data in various formats for direct download, scanner information, and activity history, etc.

In the quality control interface the user can rate both raw images and processed output. The system uses a multi rater approach, recording ratings of all authorized users separately. For quick inspection theHiveDB will create quality control images for every image or image transform visible through the web interface image library (**Figure 5**). However, images and image transforms are accessible directly in various formats or may be transferred to another resource for in-depth inspection and quality control. For instance a user may push nifti format native images for an entire project to a workstation instead of downloading them one by one in order to perform quality control. This approach allows the raters to use their preferred tools and image formats for quality evaluation. For DTI or BOLD data for instance external software is essential to perform quality control. Those images can be evaluated on a dedicated quality control station (e.g., using DTI Prep) and the results uploaded as a spreadsheet containing the image UUID as an identifier.

Quality control information can later be retrieved when querying the database. For example image processing for multiple images of varying quality can be compared to assess the impact of image artifacts and overall quality on processing output. Criteria for image QC for structural MRI image analysis pipelines have previously been published (Simmons et al., 2009, 2011).

### DATA PROCESSING AND WORKFLOW

Computing resources (i.e., physical or virtual machines) can be managed through theHiveDB activity system. A compute resource is registered by means of providing a host (i.e., IP address, hostname) and ssh login credentials (i.e., username and password). The user registering the compute resource will be considered its owner by theHiveDB. For theHiveDB to actually utilize the resource, a resource purpose (e.g., processing resource, project server or dropbox) needs to be assigned. Choosing “processing resource” will prompt for input and output paths to be registered. At this point an “activity instance” can be created. Simplified an “activity instance” corresponds to the invocation of a specific command/program on that resource (multiple instances can be created with different parameters and environment settings passed to the command). By means of granting access to projects the resource owner manages which project data can be transferred to the resource and processed as defined in the “activity instance.” TheHiveDB instance will act on behalf of the user login registered by the resource owner. To optimize resource use through collaboration without compromising processing speed, requests for external projects (i.e., from other HiveDB instances) can be assigned to a separate grid engine queue.

Within this collaborative ecosystem algorithm developers can create versioned virtual machines capable of running their tools using the cloud (e.g., Amazon cloud ec2)^11^. TheHiveDB users can run instances of these virtual machines and assign them to projects in order to take advantage of these algorithms. To illustrate the potential of this approach, consider the example of a virtual machine created in the cloud using a standard Linux installation with grid engine enabled and the Freesurfer 5.3 package added. At this point the compute resource can be registered by its owner in any HiveDB instance. Furthermore the resource owner can register activity instances and assign them to projects in order to authorize them to use the resource. An activity instance defines the algorithm or activity to be used (e.g., Freesurfer version 5.3), the activity parameters to be used and the projects authorized to request it. Activity parameters are command line arguments to be passed to the command to be executed (e.g., Freesurfer can run with “-all-mprage-nuintensitycor-3 T” for a project with 3 T imaging data and with a basic exploratory argument like “-recon1” for a second project).

While the above can also be achieved with a conventional physical server, the cloud approach has a number of advantages. Apart from minimal storage costs a cloud image only incurs cost to the user when it is used. It doesn’t physically break down and its hardware can improve over time as newer technology is made available by the cloud provider. When newer versions of algorithms are released images of previous versions may be kept for ongoing projects still requiring them. This is especially relevant when different versions of software packages cannot be installed on the same physical system.

### PROVENANCE AND META-DATA MANAGEMENT

**FIGURE 6:**
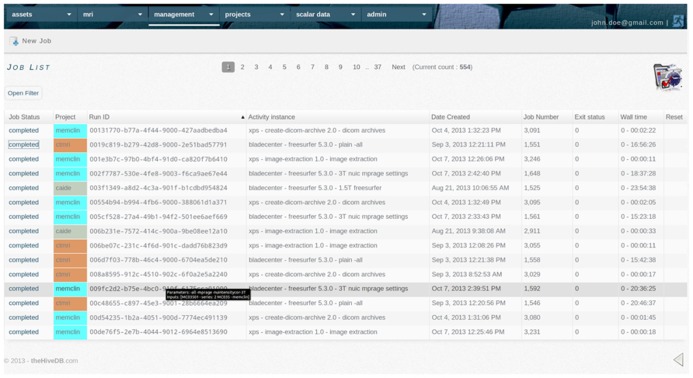
**The activity system keeps a track record of all activities performed by the system or requested by users.** It communicates with remote resources for status updates and retrieves output collections automatically. Accounting information is compiled using the grid scheduler’s accounting output.

Imaging source data is fully documented as described in the “DICOM management, storage, and compression” section. All assets produced or derived within the database system are traceable using the job sub-system (**Figure 6**). Every activity within theHiveDB consumes input and produces an output collection (i.e., a compressed archive file containing the individual results obtained from an image processing activity). The output of any activity is considered to be a collection containing at least one item. If members of an output collection have been defined they can be extracted automatically by the system (e.g., tissue classification result image obtained from a processing pipeline).

This concept provides full traceability of newly generated data, transparency for all project members, and an ever current inventory to assess project progress. A fully-fledged activity system is a prerequisite for enabling advanced processing for extremely large amounts of imaging data. Algorithm comparison with regard to stability across versions and vulnerability to image artifacts can be performed. For instance, all images obtained from an individual during a MRI session can be processed individually or in combination. Additionally multiple versions of the processing algorithm can be used and results compared.

## USE CASE

To illustrate the application of theHiveDB consider the scenario where researchers wish to download and subsequently analyze raw data from the ADNI study, a large North American study which includes 1.5 T MRI, 3 T MRI, FDG, and amyloid PET, together with CSF samples, clinical, cognitive, neuropsychological, and genetic data.

The raw imaging data for the study can be downloaded from the LONI distribution system^12^ in the form of collections of raw DICOM data with xml files for each image series. Additionally data from other modalities such as cognitive tests, demographic information, genetic data, and CSF data can be downloaded in tabular form.

The user has the following requirements for image databasing and analysis:

• Users need to be able to perform operations like tabular data import and imaging data upload through the web interface.

• No alterations of the database structure (i.e., adding tables) should be needed for newly added variables or results derived using imaging data processing algorithms.

• Tabular data import should be possible instead of data re-entry through a web interface.

• Users need to be able to deactivate projects. Regardless of a user’s authorization to see data (e.g., has access to 40 projects) the user needs to be able to activate only those of current interest.

• Data needs to be accessible directly by project members via the database web interface without the need for an intermediary (e.g., a database manager retrieving data) when image processing is desired.

• The system needs to support multiple image file formats as the inputs and outputs of different image analysis pipelines and manage data effectively as opposed to merely registering file pointers for a single file format.

• Processing of as many images as available for any given timepoint using algorithms and pipelines available is required, for example processing the two T1 volume images acquired as part of ADNI-1 and ADNI-2.

• Integration of existing infrastructure and processing capabilities with automated processing as triggered by the database system.

• As new versions of image analysis pipelines become available it must be possible to maintain multiple versions of both the algorithms and the results of the analysis pipelines (for example Freesurfer versions 5.1 and 5.3).

TheHiveDB was designed to provide the feature set of similar distribution and collection systems in the neuroimaging domain, but extending them to a more complete framework with the above requirements in mind.

### SCALAR DATA IMPORT

TheHiveDB allows for collation of existing data by simply uploading spreadsheets with scalar data. Variables are grouped into variable collections. The import is governed by conventions. Using existing collection names will add data to collections using the first line as field names. If variables are identified as members of the same collection they are queryable across projects (e.g., if Mini-mental state examination MMSE data is always imported using the same field names).

Variables can be imported (see **Figure 3**) and may later be queried at these three levels:

1. Describing individuals (permanently) like some genetic data or gender.

2. Describing individuals at specific timepoints (e.g., clinical or cognitive tests).

3. Describing assets obtained to assess individuals at specific timepoints (e.g., MR images or volume results from processing pipelines).

Via theHiveDB web interface a user creates a new project “adni” and creates or assigns an existing compute resource for project data (i.e., project server). Disk space of the project server will be used as primary location for all project data assigned to this project. The user registers timepoints (i.e., adni visit identifiers) and defaults for desired image format conversion. For this example DICOM and nifti are chosen as available formats.

The user downloads a list of ADNI study participants and creates a spreadsheet containing the following fields: project, SiteId. Gender, and DateOfBirth. Gender and DateOfBirth are not mandatory, but may be provided. The file is renamed to “adni.individuals.list.csv” and uploaded. All individuals are now registered and assigned to the “adni” project. Following the examples outlined in **Figure 3** more data describing the individual permanently or describing the individual at a specific timepoint may be uploaded.

### IMAGING DATA UPLOAD PREPARATION

Raw ADNI imaging data is downloaded via the LONI distribution system, resulting in a folder structure based on the ADNI series identifier. Auxiliary xml files with summary information about the individual, series and assignment to a visit identifier will be found at the top level of the folder structure.

For smaller projects data would be uploaded directly to theHiveDB web interface marked as anonymized and assigned to projects, individuals, and timepoints. In view of the amount of data downloaded [ADNI MP-RAGE (T1) data occupies 400 GB of disk space] and since data is known to be anonymized, an alternative route to DICOM archive creation is used. Based on information from the ADNI xml files a spreadsheet containing the following columns is created:

• Project (i.e., “adni”)

• Individual (i.e., the PatientID as found in DICOM header or xml file)

• TimePoint (i.e., the visit identifier found in the xml file)

• SourceLocation (i.e., the location data has been downloaded to)

• TargetLocation (i.e., the location where the DicomArchive and descriptor file is to be created. If the project server location is available the user may choose it to avoid data transfers.)

TheHiveDB provides a convenience function for large data collections. Upon upload the spreadsheet (in this case ~16,000 rows) will be converted into a job script, which can be submitted to the queue for HiveDB DICOM archive creation. This activity requires no connection to the HiveDB instance and can run directly on the Linux machine already hosting the downloaded data. UUIDs are computed using the same mechanism as within theHiveDB and for every folder containing DICOM data a compressed DICOM archive and a supplementary descriptor file in JSON format^13^ is created. For the above example the procedure takes on average 5 s per folder. Within 3 h on a desktop machine (eight cores) this process transforms almost three million single DICOM slices into about 16,000 completely documented and compressed DICOM archives.

### DATA IMPORT AND ORGANIZATION

The descriptor files are subsequently uploaded via theHiveDB web interface resulting in DICOM archives being automatically created and assigned to project, individuals, and timepoints. The user defines at least one scanning protocol for the “adni” project, such that the system can automatically identify T1 data. After a test search the user confirms the protocol as valid leading to automatic extraction of all MR image assets and the creation of downloadable image files in DICOM and nifti format. All data is now available to project members through the web interface.

### DATA PROCESSING

The user registers a processing resource (i.e., an existing cluster the user has access to) and defines two activity instances through the web interface (One instance with parameters to be used for 1.5 T data labeled “Freesurfer-5.1 1.5 T” and another one for 3.0 T data labeled “Freesurfer-5.1 3.0 T”).

The user now selects the current session preferences panel and deactivates all projects other than “adni.” In the image library the user now searches for T1, 1.5 T, and timepoint M00 (i.e., baseline) data and chooses “Freesurfer-5.1 1.5 T” as activity to apply to all elements found, followed by the same procedure for 3.0 T data. Standard processing time for Freesurfer is in the vicinity of 16 h per image. Processing all 16,000 images on the 100 core cluster currently providing processing for the production database will take about 3 months. For this reason timepoints will be submitted in sequence in order to start analysis on data as it becomes available. The user may now log off.

TheHiveDB will create job files (using computed UUIDs for names), transfer inputs to the processing cluster and submit jobs. It will monitor the queue and upon job completion retrieve an output collection (a tar file) containing the results for every single job.

If new versions of the pipeline (e.g., Freesurfer 5.3) become available, the creation of additional activity instances is required. The steps above are repeated with the new version of the pipeline. Since the activity version is part of the computation of UUIDs, new unique identifiers for outputs will be provided by the system.

The Freesurfer pipeline outputs a multitude of different measures (Fjell et al., 2009; Walhovd et al., 2011; Westman et al., 2013), which need to be queried and combined for analysis with data from other modalities. Since Freesurfer is directly supported by the database, volume extraction will be performed automatically and all volumes will be registered in a variable collection labeled Freesurfer-5.1. The user may now query those volumes in conjunction with other data uploaded via tabular data import. If the user produces additional measures using external methods to compute scalar values those may be uploaded following conventions depicted in **Figure 3**. TheHiveDB aggregates data from these different modalities automatically and combines it with image processing results, such that research problems can be addressed without the need to manually manage and merge spreadsheets.

## DISCUSSION

### RELATED WORK

TheHiveDB has been developed to advance imaging efforts in a context where more and more data is available to researchers either by means of in house acquisition or more frequently by means of collaboration. The latter includes the growing number of publicly available collections of imaging data such as the ADNI; Jack et al., 2008; Weiner et al., 2010) and AddNeuroMed (Lovestone et al., 2009, 2007).

Most of these collections use a distribution system (such as the LONI ADNI archive)^14^ to disseminate data. In these systems assets (the raw imaging data) and associated data from other modalities are readily accessible and frequently processed data (output collections) can also be downloaded. The LONI image data archive provides scalar data organized into spreadsheets. An accompanying data dictionary helps clarify the meaning of variable names contained in these spreadsheets. For any given group of variables (typically a questionnaire or the scalar results of some processing or other analysis of data) spreadsheets can be downloaded. It is up to the researcher to match data from different cohorts (Westman et al., 2011b) or modalities (Westman et al., 2012) prior to any data analysis being undertaken using the raw data and images. Ever changing spreadsheets have to be organized, merged, and maintained. The creation of subsets of data to investigate specific research questions remains a cumbersome process.

Other systems like the LORIS system (Das et al., 2012) focus on scalar data collection for relatively homogeneous ongoing studies. The LORIS system needs to be customized at the database structural level, before its web interface can be used as a data entry system by participating sites. Each addition of tabular data implies a change to the database structure to store data for newly added variables. While the LORIS query interface is able to match some MRI data to clinical variables, the imaging component remains an afterthought due to the system’s architectural conception as scalar data entry system. The LORIS web interface does not allow distribution of data directly as file data is only referenced in the database and solely accessible via command line interfaces on servers hosting the actual data.

For handling ongoing data collection and data entry the REDCap (Obeid et al., 2013) system appears to be a more feature complete and convenient system. REDCap is designed to comply with HIPAA regulations and can be quickly adjusted to cover all aspects of research data capture.

The LONI pipeline (Dinov et al., 2010) provides a collection of neuroimaging tools for computational scientists. It allows for workflow creation and execution via Pipeline Web Start (PWS)^15^.

The XNAT (Marcus et al., 2007) system and its Python client library PyXNAT (Schwartz et al., 2012) represent the web services approach to neuroimaging databases. Neuroimaging data is modeled through XML schemas and a representational state transfer Application Program Interface (REST API) allows software developers to programmatically interact with the database system.

Research labs can struggle with how to organize the ever growing collections of data. Most neuroimaging databases consequently provide a container based approach with a more or less predefined structure to organize data. This approach works well to organize data as long as the data stays within the realm of control of the database system. A user who downloads a set of image files to perform processing temporarily breaks the way data is structured in the database. If files have no unique identifiers or can be identified by means of header tags or md5sums the interaction of the researcher with the database system is rapidly disturbed. Data needs to be reorganized and the database needs to be updated with newly created results. Unfortunately this implies frequent changes to the actual database structure and/or creation of XML schemata.

Most of the above mentioned database systems are designed as containers or data inventories. The container approach works well for data entry systems where the size of a prospective study warrants the effort of customization, but they are frequently limited to tabular data collection. The other approaches require the user to interact programmatically with the database system to retrieve data and repopulate with results.

TheHiveDB goes beyond these approaches. It offers ways to organize data beyond simple storage. Imaging data assets are enhanced with features to simplify the researcher’s interaction with the data (See sections “DICOM management, storage, and compression” and “Images”). While programmatically interacting with theHiveDB is an option for advanced users (theHiveDB is a RESTful resource) the framework aims to accompany and support the researcher in daily activities and explorations. Standard activities can be automatically performed for any new project with existing resources and new activities can be explored with the help of the system. All assets remain identifiable within and outside the system. Even for external activities the identifier creation keeps expected results traceable. This way manual steps or external resources for free image processing can be integrated.

TheHiveDB implements the main ideas of other activity and workflow systems. Tools and algorithms are available to the researcher and can be applied to available data. To warrant consistency without compromising progress theHiveDB requires all activities to be versioned.

The primary shortcoming of some neuroimaging frameworks is their insufficient support for file data (assets). Neuroimaging research is an active field. In order to progress imaging assets need to be available and accessible to those working with them. The unique identifiers within theHiveDB constitute tracking or serial numbers for assets. The web interface acts like a tracking system providing appropriate information. For images this may be the scanner or protocol used or assessments of image quality. For output from any activity the entire process leading to its generation is traceable. The system is not designed to force all data into a container. It encourages the interaction with the researchers by letting them experiment with assets to perform activities not (yet) supported by the system. It even provides the possibility to re-integrate results by allowing for external activities where the user needs to provide the activity output by means of uploading it, using the identifier provided by the system.

Interacting programmatically with theHiveDB API remains a possibility for the so inclined power user, but it is not a requirement for researchers. The ability to voluntarily disable access to projects throughout the system can greatly simplify the researcher’s day to day interaction with the system. It can be frustrating to always have to set additional filters in order not to be exposed to all data one is authorized to see.

## FUTURE DEVELOPMENT

TheHiveDB has been conceived and developed as a data aggregation system. While it currently supports scalar data import, it would be desirable for theHiveDB to interface directly with clinical data entry systems. Especially with systems allowing non-programmers to quickly create forms for tabular data collection like the REDCap application.

ThHiveDB’s activity system supports activity creation based on asset types. While it is presently only used for image processing it would be conceivable to integrate workflows for other types of data (e.g., by supporting genetic data processing).

TheHiveDB allows users to directly access MR images in their preferred format to be visualized for quality control purposes. While we favor direct access to images in user definable formats and the possibility to push entire collections to dedicated quality control stations, the inclusion of a web based viewer with 3D capabilities may be desirable for some users. The integration of imageJ^16^ could provide additional convenience to users in this regard. Nielsen’s heuristics have influenced the design and development of theHiveDB and it has been developed in continuous interaction with future users. However, a formal evaluation of the system would be desirable.

While some architectural design elements might hint toward a federated database system currently only data exchange and migration/fusion is planned. Data ownership concerns and the protected nature of imaging database as discussed in the “Data access, permissions, and ownership” section make this a more likely scenario.

## CONCLUSION

At the topological level theHiveDB provides the integration of different components – a solid database engine combined with secure data store and an activity system for data processing purposes. The application is flexible to be adapted to individual requirements and available resources without the need to customize its database tables and structure.

TheHiveDB provides extensive cross domain integration. For tabular/scalar data, convention based import (i.e., using specific column arrangements) allows for swift integration of data already available in spreadsheets or textual form.

The asset management system provides support tailored to the particular needs of brain imaging projects. But what is more, it is also capable of integrating newly defined asset types. The generation of unique identifiers extends to any type of uploaded data and provides data integrity verification and management with storage, transfer, backup, and availability. This approach clears the way for integration of imaging workflow with other types of workflow based on custom asset types.

On an architectural level theHiveDB is capable of integrating distributed systems. Each “HiveDB” has its own unique ID. Frequently individual research groups will have their own HiveDB instance (see **Figure 4A**), but share resources for activities (i.e., data processing). Additionally cloud resources can be enabled by algorithm developers to be used by those instances of theHiveDB. Project data will in most cases be stored on local resources, but long term cloud backup (e.g., Amazon glacier) for both raw imaging data and processed output is another viable option.

TheHiveDB represents another step toward creating a complete neuroimaging research framework. It provides easy access to data just like traditional distribution systems and offers the convenience of multi modal querying.

A key aim of theHiveDB is to enable collaborations. It does so by providing a framework for neuroimaging projects based on sound data management, organization, and documentation. Upon that base rests an activity system allowing for automation and resource sharing while ensuring full traceability of activities and outputs. With its asset management and activity system it establishes a powerful ecosystem for collaborative work and resource sharing in continuous interaction with the researcher.

The inclusion of standard communication protocols and job schedulers eliminates the need for a human data manager needed in most of the other systems available to date. TheHiveDB knows where project data is supposed to be stored and where it can be processed. It is capable of performing its own data transfers and request activities/processing to that effect.

The system has been designed to interact with the researcher in a (human) way that does not require the acquisition of database query language skills or programming proficiency.

## AUTHOR CONTRIBUTIONS

J-Sebastian Muehlboeck, Eric Westman, and Andrew Simmons all contributed to the concept of TheHiveDB, J-Sebastian Muehlboeck is the programmer and developer of the system. J-Sebastian Muehlboeck, Eric Westman, and Andrew Simmons all contributed to the writing and editing of the manuscript.

## Conflict of Interest Statement

J-Sebastian Muehlboeck is lead developer and president of J-S Muehlboeck Inc. Eric Westman and Andrew Simmons have no conflict of interest.

## Footnotes

1 http://www.hibernate.org/

2 http://www.mysql.com/why-mysql/topreasons.html

3 http://quartz-scheduler.org/

4 http://www.snailbook.com/protocols.html

5 http://packages.debian.org/wheezy/gridengine-client

6 http://medical.nema.org/standard.html

7 http://nifti.nimh.nih.gov/

8 http://www.nitrc.org/projects/minc/

9 http://surfer.nmr.mgh.harvard.edu/

10 http://fsl.fmrib.ox.ac.uk/fsl/fslwiki/

11 http://aws.amazon.com/ec2/

12 https://ida.loni.usc.edu/login.jsp

13 http://www.json.org/

14 http://adni.loni.usc.edu/data-samples/access-data/

15 http://pipeline.loni.usc.edu/products-services/pws/

16 http://rsb.info.nih.gov/ij/index.html
